# Executive functioning, ADHD symptoms and resting state functional connectivity in children with perinatal stroke

**DOI:** 10.1007/s11682-023-00827-w

**Published:** 2023-12-01

**Authors:** Suraya Meghji, Alicia J. Hilderley, Kara Murias, Brian L. Brooks, John Andersen, Darcy Fehlings, Nomazulu Dlamini, Adam Kirton, Helen L. Carlson

**Affiliations:** 1 Calgary Pediatric Stroke Program, Alberta Children’s Hospital, 28 Oki Drive NW, Calgary, AB Canada; 2 Alberta Children’s Hospital Research Institute, 28 Oki Drive NW, Calgary, AB Canada; 3 Department of Pediatrics, Cumming School of Medicine, University of Calgary, Calgary, AB Canada; 4 Hotchkiss Brain Institute, University of Calgary, Calgary, AB Canada; 5 Department of Clinical Neurosciences, University of Calgary, Calgary, AB Canada; 6 Neurosciences Program, Alberta Children’s Hospital, Calgary, AB Canada; 7 Department of Psychology, University of Calgary, Calgary, AB Canada; 8 Department of Pediatrics, University of Alberta, Edmonton, AB Canada; 9 Department of Pediatrics, University of Toronto, Toronto, ON Canada; 10 Children’s Stroke Program, Division of Neurology, Hospital for Sick Children, Toronto, ON Canada; 11 Department of Radiology, University of Calgary, Calgary, AB Canada

**Keywords:** Perinatal stroke, Functional connectivity, ADHD, Executive function, Cognition, Default mode network

## Abstract

Perinatal stroke describes a group of focal, vascular brain injuries that occur early in development, often resulting in lifelong disability. Two types of perinatal stroke predominate, arterial ischemic stroke (AIS) and periventricular venous infarction (PVI). Though perinatal stroke is typically considered a motor disorder, other comorbidities commonly exist including attention-deficit hyperactivity disorder (ADHD) and deficits in executive function. Rates of ADHD symptoms are higher in children with perinatal stroke and deficits in executive function may also occur but underlying mechanisms are not known. We measured resting state functional connectivity in children with perinatal stroke using previously established dorsal attention, frontoparietal, and default mode network seeds. Associations with parental ratings of executive function and ADHD symptoms were examined. A total of 120 participants aged 6–19 years [AIS *N* = 31; PVI *N* = 30; Controls *N* = 59] were recruited. In comparison to typically developing peers, both the AIS and PVI groups showed lower intra- and inter-hemispheric functional connectivity values in the networks investigated. Group differences in between-network connectivity were also demonstrated, showing weaker anticorrelations between task-positive (frontoparietal and dorsal attention) and task-negative (default mode) networks in stroke groups compared to controls. Both within-network and between-network functional connectivity values were highly associated with parental reports of executive function and ADHD symptoms. These results suggest that differences in functional connectivity exist both within and between networks after perinatal stroke, the degree of which is associated with ADHD symptoms and executive function.

**Supplementary Information:**

The online version contains supplementary material available at 10.1007/s11682-023-00827-w.

## Introduction

Perinatal stroke describes a group of focal, vascular brain injuries that occur early in development with a population-based birth prevalence of ~ 1 in 1100 (Dunbar & Kirton, 2019). The perinatal period is the most common pediatric period for stroke; however, the cause and risk factors are still unclear (Dunbar & Kirton, 2019). Perinatal stroke causes the majority of hemiparetic cerebral palsy and a lifetime of motor disability with no known prevention strategies (Dunbar & Kirton, 2019; Kirton & deVeber, 2013; Lee et al., 2005; Wu et al., 2004). Two types of perinatal stroke predominate, arterial ischemic stroke (AIS) and periventricular venous infarction (PVI) (Dunbar & Kirton, 2019; Fehlings et al., 2021). AIS is a focal area of infarction corresponding to arterial territories (commonly the middle cerebral artery) and can lead to extensive cortical and subcortical damage. PVI results from a germinal matrix hemorrhage occurring before 34 weeks gestation, resulting in infarction of the periventricular white matter. As focal, unilateral injuries with defined timing, perinatal stroke is an ideal human model to study developmental plasticity (Kirton et al., 2021).

Though perinatal stroke is typically considered a motor disorder, other comorbidities commonly occur including attention-deficit hyperactivity disorder (ADHD) symptoms and deficits of executive function. Diagnoses of ADHD appear to be higher in children with perinatal stroke (19–35%) as compared to the general population (7%) (Bosenbark et al., 2018; Craig et al., 2018; Max et al., 2005; Thomas et al., 2015). ADHD is characterised by difficulty concentrating, poor inhibitory control, overactivity and impulsivity, disrupting activities of daily living, academic achievement and mental health (Bosenbark et al., 2018; Gräf et al., 2019; Pingault et al., 2014; Shoval et al., 2021). Deficits in executive function, characterized by poor attentional control, cognitive flexibility and problem solving behaviour, have been documented in children with perinatal stroke (Bosenbark et al., 2018; Kirton & deVeber, 2013; Murias et al., 2014). Stroke-induced changes in the development of cortical networks mediating cognition may underlie these differences, but there is currently a paucity of evidence.

Neuroimaging techniques like resting state (RS) functional magnetic resonance imaging (fMRI) may help elucidate possible underlying disruptions of network connectivity. RS-fMRI measures low frequency fluctuations in the blood-oxygen level dependent (BOLD) signal in the resting brain and quantifies functional connectivity (Biswal et al., 1995; Fox & Raichle, 2007). Alterations of connectivity within the motor network have been observed in perinatal stroke (Al Harrach et al., 2021; Carlson et al., 2020; Manning et al., 2015, 2016; Saunders et al., 2019; Woodward et al., 2019), however, there has been little investigation into how alterations in resting state networks might mediate cognition (Carlson et al., 2019; Ilves et al., 2016).

Numerous resting state networks, including the dorsal attention (DAN), frontoparietal (FPN), and default mode (DMN) networks have been identified (Fair et al., 2008; Fox & Raichle, 2007; Fox et al., 2006; Greicius et al., 2003; Power et al., 2010; Raichle et al., 2001; Vincent et al., 2008; Yeo et al., 2011), the characteristics of which are associated with ADHD symptoms and executive function (Fair et al., 2012; Fassbender et al., 2009; Francx et al., 2015; Gao et al., 2019; Lin et al., 2015; Sanefuji et al., 2017; von Rhein et al., 2016). In addition to differences *within* functional networks, the complex interplay *between* networks undergoes significant development during childhood and adolescence (Dwyer et al., 2014; Luna et al., 2015; Satterthwaite et al., 2013). Resting state networks have been shown to be anticorrelated such that task-positive networks (DAN, FPN) and task-negative networks (DMN) show strong inverse functional connectivity with each other (Fox et al., 2005). During development, this complex relationship may be dynamic and context-dependent (Dwyer et al., 2014) and may also be altered in children with ADHD (Gao et al., 2019).

Despite the paucity of studies investigating imaging biomarkers of cognition after perinatal stroke, interesting preliminary evidence in a group of children with AIS has shown higher functional connectivity in the posterior precuneus area of the DMN compared to controls (Ilves et al., 2016). Most of the children with AIS also showed poorer cognitive function compared to peers as assessed by a comprehensive clinical evaluation. This suggests a potential association between levels of functional connectivity and cognitive function though this association was not directly tested. In the same study, for a group of children with PVI, cognitive abilities were largely in the normal range and functional connectivity was more similar to controls. Whether deficits in cognition are directly associated with differences in resting state networks has yet to be extensively investigated but this initial work has elegantly established clinical relevance.

We explored functional connectivity within and between the FPN, DAN, and DMN networks and associations with executive function and ADHD symptoms. We hypothesized that because of the more extensive cortical lesions in children with AIS, they would have lower resting state functional connectivity (FC) within the FPN, DAN and DMN networks compared to both PVI and typically-developing control (TDC) groups, who would be more similar to each other. We further hypothesized that functional connectivity within (and between) these networks would be associated with cognitive scores such that lower connectivity would be associated with poorer function.

## Methods

### Participants

Participants were recruited via two urban Canadian centres as part of ongoing motor rehabilitation trials: the first from a population-based research cohort (the Alberta Perinatal Stroke Project, APSP)(Cole et al., 2017) and the second from Holland-Bloorview Kids Rehabilitation Hospital. Inclusion criteria for both sites were: (1) MRI-confirmed unilateral perinatal stroke (AIS or PVI) congruent with validated criteria (Kirton et al., 2008); (2) hemiparetic cerebral palsy as determined by a Pediatric Stroke Outcome Measure (PSOM) motor score of > 0.5 (Kitchen et al., 2012); (3) term birth (> 36 weeks), and (4) current age of 6–19 years. Individuals with extensive lesion damage precluding analysis, bilateral strokes, multiple strokes, or other neurological diagnoses were excluded. Due to the presence of contralateral hemiparesis the patients were considered “best-handed” using the hand on the ipsilesional side as their dominant hand.

Typically developing controls (TDC) were recruited through a volunteer-based healthy control program at the first centre. Inclusion criteria were: 1) self-reported right-handedness; 2) ages 6–19 years; 3) no neurological or psychiatric conditions; and 4) no MRI contraindications. Informed, written parental consent and participant assent were obtained in accordance with the local Research Ethics Boards which approved this study.

### Imaging

Neuroimaging was performed using two 3.0 Tesla scanners. The Alberta Children’s Hospital Diagnostic Imaging Suite utilized a General Electric MR750w MRI scanner with a 32-channel head coil. T1-weighted fast spoiled gradient echo images were acquired in the axial plane [166 contiguous slices; voxels = 1.0 mm isotropic; repetition time (TR) = 8.5 ms; echo time (TE) = 3.2 ms]. The Holland-Bloorview cohort used a Siemens Prisma scanner with a 20-channel head coil to acquire 3D Magnetization-Prepared Rapid Gradient-Echo (MPRAGE) T1-weighted images obtained in the sagittal plane [192 contiguous slices, voxel size = 1 mm isotropic, TR/TE = 2.3 s/3.0 ms]. On both scanners, RS-fMRI sequences were obtained using 150 T2*-weighted whole brain echo planar volumes (36 contiguous axial slices; voxels = 3.6 mm isotropic; TR/TE = 2000/30 ms; duration 5 min). During RS-fMRI scanning participants were asked to fixate on a central cross. Differences in FC measurements between scanners were statistically tested and scanner was also used as a covariate.

### Lesion mapping

Lesion sizes were quantified by manually demarcating primary lesions in MRIcron (Rorden et al., 2007) via the 3D fill and other drawing tools on the T1-weighted anatomical image using methods similar to Liew et al. (Liew et al., 2018). This demarcation was done based on image intensity by a pediatric neuroimager with extensive experience in delimiting stroke lesions. Lesion masks were then smoothed using the volume of interest smoothing tool (2 mm kernel, threshold = 0.5) and volumes containing non-zero voxels were extracted. PVI lesion sizes were calculated by first demarcating each ventricle as above, applying the smoothing kernel, and finally subtracting the ventricle in the lesioned hemisphere from the non-lesioned hemisphere. Lesion masks were subsequently warped into MNI152 standard space using the Normalize function in SPM12 (Statistical Parametric Mapping, UCL, London) running within Matlab 2019a (The Mathworks, Natick, Mass., USA) for visualization.

### Image preprocessing

Because all controls were right-handed, the left hemisphere was considered the dominant hemisphere in this group. MR images for patients were reoriented such that all lesions were located on the right side to allow for comparisons between the non-lesioned hemisphere in participants with stroke and the dominant (left) hemisphere in controls. Conversely, lesioned hemispheres in those with stroke were compared with non-dominant hemisphere (right) in the TDC group. Thus, hemispheres are subsequently referred to as lesioned (non-dominant in TDC) and non-lesioned (dominant in TDC) rather than right and left.

FC analyses were conducted on RS-fMRI sequences using the Functional Connectivity Toolbox (CONN) (Whitfield-Gabrieli & Nieto-Castanon, 2012), an SPM12 extension. Utilization of the CONN pipeline allowed for standard preprocessing, which included slice time correction, realignment and co-registration. Calculation of head motion parameters was performed using the Artifact Repair Toolbox (ART) (Whitfield-Gabrieli et al., 2011) and volumes that exceeded 0.9 mm of translational head motion or had a global signal z-score > 5 were identified and de-weighted (i.e., “scrubbed”) in the subsequent general linear model (GLM). Participants were required to have at least 100 volumes retained after scrubbing to be included. Segmentation of the co-registered anatomical images was done using standard SPM tissue probability maps, with lesions categorized as cerebrospinal fluid (CSF). Normalization of the images into standard Montreal Neurological Institute (MNI) space was performed using the 152-average template in CONN/SPM12. Images were reviewed slice-by-slice in the axial plane to ensure accuracy of segmentation and normalization procedures. Images were smoothed with 6mm^3^ full-width at half-maximum (FWHM) Gaussian kernel. A GLM subsequently de-weighted previously identified volumes from the head motion correction step as well as CSF and WM signal time courses.

### Seed-to-seed analyses

**Fig. 1:**
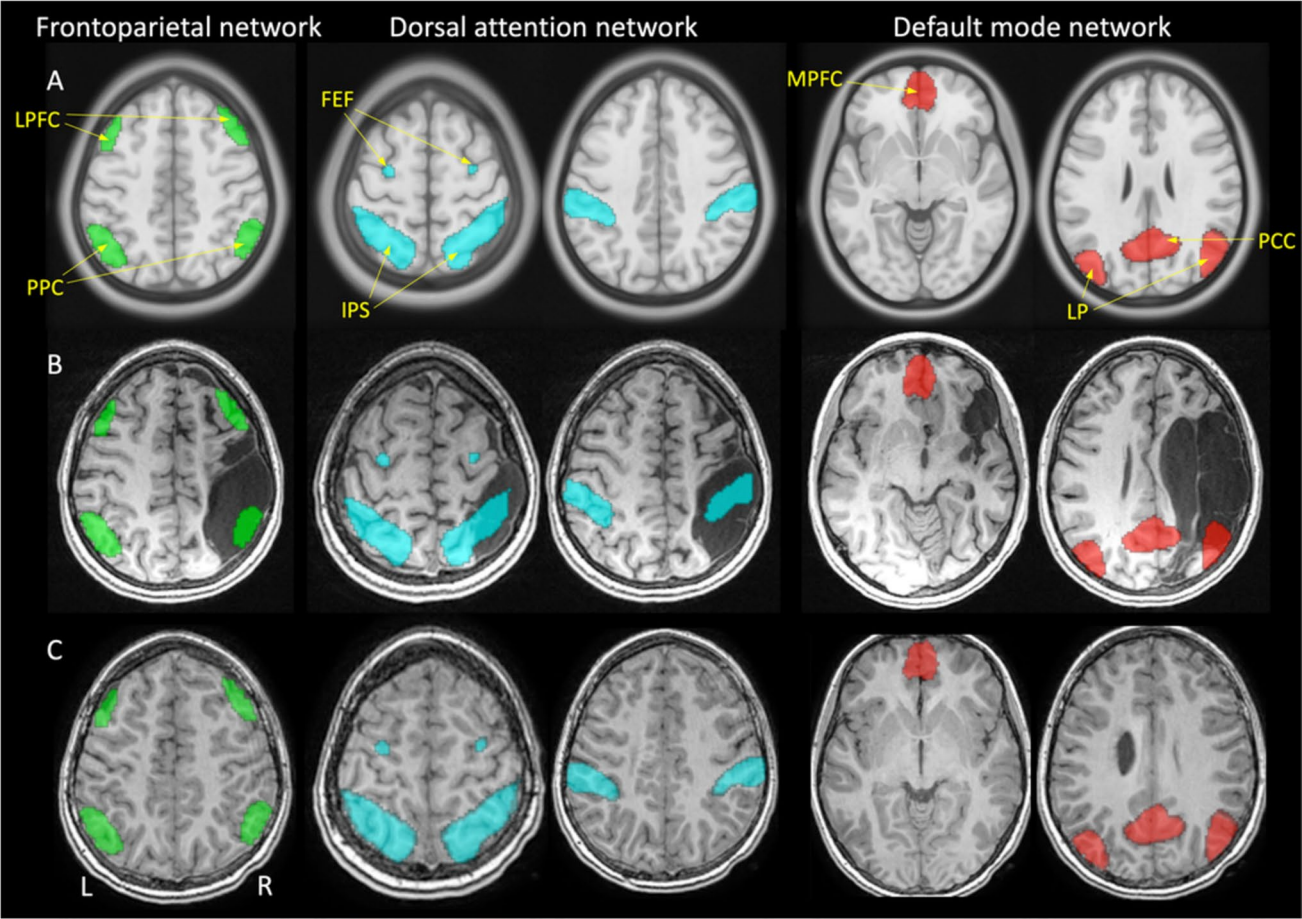
Four seeds from each of the frontoparietal, dorsal attention, and default mode networks are displayed on (**A**) a template image in MNI space, and normalized images from two individuals with either an (**B**) arterial ischemic stroke (AIS) or (**C**) a periventricular venous infarction (PVI). Several seeds were subsequently excluded given that there were not enough voxels containing gray matter to reliably measure BOLD signal. Details as to how this was determined are described in methods. LPFC—lateral prefrontal cortex, PPC—posterior parietal cortex, FEF—frontal eye fields, IPS—intraparietal sulcus, MPFC—medial prefrontal cortex, LP—lateral parietal cortex, PCC—posterior cingulate cortex. All stroke lesions are on the right side of the brain images

Seed-to-seed analyses quantified FC within and between networks. Seeds were defined via an independent component analysis (ICA) performed by the developers of CONN using resting state data from the Human Connectome Project (*N* = 497 participants) and are provided as part of the CONN distribution (Whitfield-Gabrieli & Nieto-Castanon, 2012). Four seeds were identified from each of the FPN, DAN and DMN networks (Fig. 1). FPN seeds were bilateral lateral prefrontal cortex (LPFC) and posterior parietal cortex (PPC). DAN seeds were the bilateral frontal eye fields (FEF) and the intraparietal sulcus (IPS). DMN seeds were the medial prefrontal cortex (MPFC), bilateral lateral parietal cortex (LP) and the posterior cingulate cortex (PCC). For bilateral structures, the nomenclature of lesioned (Les) and non-lesioned (NonLes) is used to distinguish hemispheres (e.g., LPFC_Les_ and LPFC_NonLes_). Fisher-transformed Pearson correlation coefficients quantifying the temporal cross correlation of low frequency BOLD fluctuations between each seed pair were extracted for both within-network and between-network seed pairs.

For stroke patients, each seed was overlaid on the grey matter mask from the segmentation pre-processing step. The number of voxels containing grey matter (i.e., non-zero voxels) was totalled for each seed and any seeds containing fewer voxels than two standard deviations from the group mean were excluded. This process allowed for seeds spatially displaced from the lesion to be included in the analysis even if those seeds falling directly in the lesion were necessarily excluded. This analysis also attempted to ensure adequate sampling of BOLD response in seeds partially overlapping the lesions.

### Seed-to-voxel analyses

Additional second-level seed-to-voxel analyses were performed to explore voxel-wise anticorrelations in between-network connectivity of the DMN (task-negative) with voxels in task-positive networks. The medial prefrontal cortex (MPFC) seed from the DMN was used to measure group mean FC with all other voxels in the brain for each participant group (AIS, PVI, TDC). This seed was selected because it was less likely to be affected by primary lesion damage in both stroke groups given the vascular territories affected by AIS and PVI (Dunbar & Kirton, 2018) and because it is a central region within the DMN known to anticorrelate with task-positive networks (Fox et al., 2005). Family-wise error (FWE) correction was employed using a threshold of p_FWE_ < 0.05 to ensure a relatively conservative multiple comparison correction (Nichols & Hayasaka, 2003). Between-group contrasts were also performed to quantify statistical differences in voxel-wise anticorrelations using this same MPFC seed and p-values were FWE corrected (group contrasts: TDC > AIS, TDC > PVI and PVI > AIS).

### ADHD symptoms and executive function

A subset of parents of children with AIS and PVI from the ACH cohort completed questionnaires measuring executive functioning (Behaviour Rating Inventory of Executive Function (BRIEF)) and ADHD (ADHD Rating Scale version 5 (ADHD-5)) as part of a standard of care clinical referral for a neuropsychological assessment. Parents of AIS and PVI participants from the Holland-Bloorview cohort and parents of the TDC participants did not complete these questionnaires.

The BRIEF is a reliable and valid questionnaire used to assess a child’s executive function behaviours and is optimized for those with learning disabilities, brain injuries, and attentional disorders (Gioia et al., 2000). The BRIEF comprises eight subscales: Inhibit, Shift, Emotional Control, Initiate, Working Memory, Plan/Organize, Organization of Material and Monitor. Subscales form two broader indices, the Behavioural Regulation Index (BRI) and Metacognition Index (MI), which are used to calculate the Global Executive Composite (GEC). The subscales are assessed through parent reports of behaviour and are expressed as T-scores (range: 20–80) in relation to an age- and sex-matched normative database.

Parental ratings of ADHD were quantified using the ADHD-5 questionnaire, a reliable clinical assessment of ADHD and related symptoms (DuPaul et al., 2016). This questionnaire consists of two subscales, hyperactivity-impulsivity and inattention and scores are expressed as percentiles (range: 0–100) in relation to an age- and sex-matched normative database. Both the BRIEF and ADHD-5 questionnaires are negatively scored such that higher scores represent poorer executive function and higher ADHD symptoms, respectively.

### Statistical analysis

Statistical analyses were conducted using R (R Core Team, 2017) and Jamovi version 1.6.23 (Jamovi, 2021). Distribution normality was determined using Shapiro–Wilk tests. Between-group equality for demographic variables was investigated with Chi-square tests (sex) and analysis of variance (ANOVA) (age) followed by post hoc pairwise comparisons. FC values were compared between scanners using Student’s t-tests. Group differences in number of volumes scrubbed were examined with a Kruskal–Wallis one-way ANOVA. Associations between age and FC were explored using Spearman’s rho. Analyses of covariance (ANCOVAs) were used for between-group (using age and scanner as covariates) and between-hemisphere comparisons of FC values followed by post-hoc independent samples t-tests where appropriate.

One-sample t-tests and Wilcoxon W tests examined whether cognitive scores differed from expected population values (BRIEF T-score = 50, ADHD percentile = 50) for each stroke group. Independent samples t-tests, or Mann–Whitney U tests as appropriate, investigated group differences between AIS and PVI for cognitive variables. To maximize statistical power for relatively small samples, AIS and PVI groups were subsequently combined into one group for the cognitive outcome analyses. Associations between FC and cognitive outcomes were explored using partial Spearman correlations (controlling for age). Where applicable, effect sizes were quantified using Cohen’s d or partial Eta squared (η^2^_p_). Multiple comparisons were corrected via the Benjamini–Hochberg False Discovery Rate (FDR) procedure using a critical value of p_FDR_ < 0.05 (Benjamini & Hochberg, 1995).

## Results

### Participants

A total of 130 participants were initially recruited. Due to excessive head motion, ten participants were excluded (7 males, 3 females). The final sample consisted of 120 participants [AIS, *N* = 31; PVI, *N* = 30; TDC, *N* = 59]. Participant group demographics are detailed in Table 1. For six AIS participants, individual seeds were removed from specific analyses due to insufficient volumes of grey matter within the seed area. The resulting sample sizes for each seed pair are included in Tables S1-5 for clarity.

**Table 1:** Demographics and cognitive function by participant group

Demographics by participant group	AIS (*N* = 31)	PVI (*N* = 30)	TDC (*N* = 59)
Mean age (SD) [range] years	13.0 (3.6) [6.6–19.5]	12.1 (3.3) [6.7–19.7]	14.2 (3.0) [6.5–19.0]
Sex [%]			
Male Female	*N* = 16 [51.6%] *N* = 15 [48.4%]	*N* = 18 [60.0%] *N* = 12 [40.0%]	*N* = 30 [50.8%] *N* = 29 [49.2%]
Stroke hemisphere (MRI) [%]			
Left Right	*N* = 21 [67.7%] *N* = 10 [32.3%]	*N* = 21 [70.0%] *N* = 9 [30.0%]	**–** **–**
Mean stroke volume (SD) cm^3^	56.6 (57.7)	7.8 (13.0)	**–**
Scanner			
GE 3 T MRI Siemens 3 T MRI	*N* = 28 [90.3%] *N* = 3 [9.7%]	*N* = 26 [86.7%] *N* = 4 [13.3%]	*N* = 59 [100%] –

*AIS* arterial ischemic stroke, *PVI* periventricular venous infarction, *TDC* typically developing controls

The three groups were comparable for sex [χ^2^_(2)_ = 0.72, *p* = 0.70] but differed slightly in age [F_(2,60)_ = 4.36, *p* = 0.02] such that the PVI group was younger than the TDC group (*p* = 0.02). No significant differences were found for FC values between scanners (all *p*-values > 0.10). The number of volumes scrubbed due to outliers was not different (χ^2^_(2)_ = 0.52, *p* = 0.77) between the three groups [mean number of volumes scrubbed (SD): AIS = 8.97 (13.7), PVI = 8.23 (13.5), TDC = 7.36 (13.4)].

### Lesion mapping

**Fig. 2:**
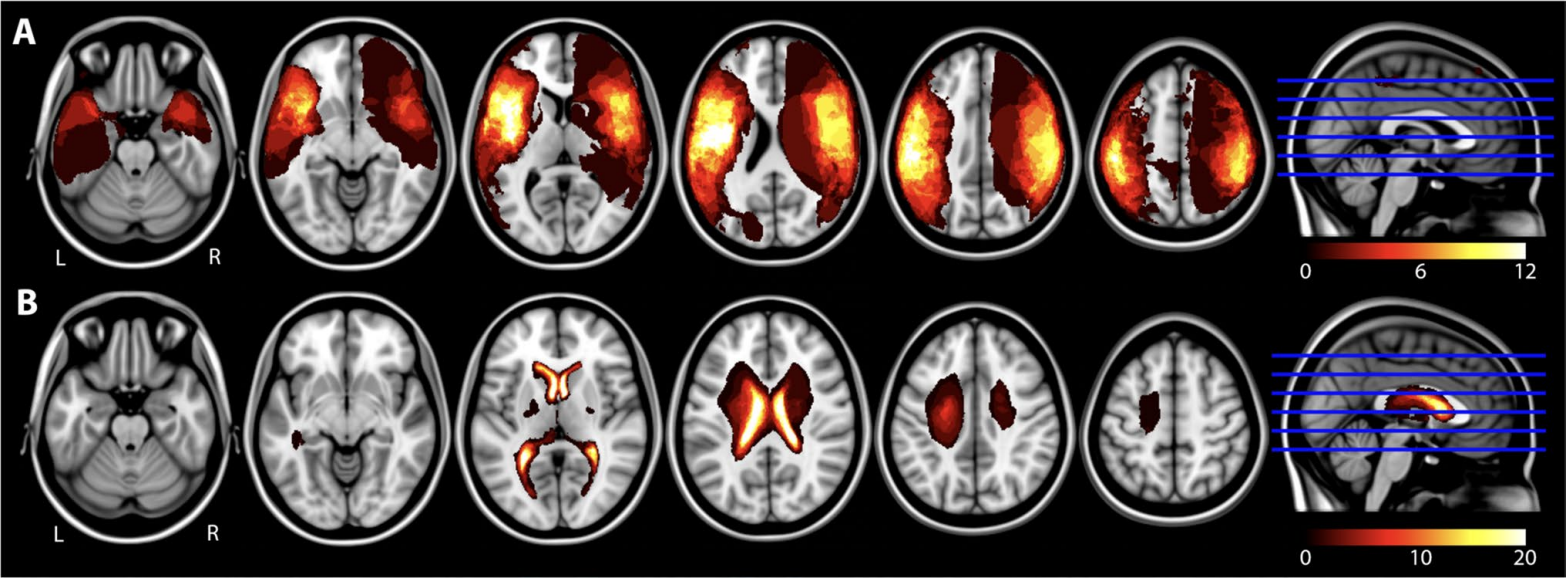
Lesion overlay maps for the (**A**) arterial ischemic stroke (AIS) and (**B**) periventricular venous infarction (PVI) patient groups. Brighter colours represent larger numbers of participants with a lesion in that brain area

Lesion overlap maps for each of the two stroke groups are illustrated in Fig. 2 where brighter areas reflect greater overlap among participants. Group lesion sizes are reported in Table 1.

### Age correlations

Age was associated with FC for some seed pairs in each of the three participant groups. For the FPN, FC for the LPFC_NonLes_-PPC_Les_ (AIS: r_s_ = 0.44, *p* = 0.03) and LPFC_NonLes_-PPC_NonLes_ (TDC: r_s_ = 0.30, *p* = 0.02) seed pairs were positively associated with age. For the DAN, FC for the FEF_NonLes_-IPS_NonLes_ (TDC r_s_ = -0.26, *p* = 0.04) and FEF_Les_-IPS_NonLes_ (PVI: r_s_ = 0.39, *p* = 0.03; TDC r_s_ = -0.29, *p* = 0.03) seed pairs were associated with age. For the default mode network, FC for the MPFC-LP_NonLes_ (TDC: r_s_ = -0.31, *p* = 0.02), MPFC-LP_Les_ (TDC: r_s_ = -0.33, *p* = 0.01), LP_NonLes_-PCC (AIS: r_s_ = -0.38, *p* = 0.04; PVI: r_s_ = -0.54, *p* = 0.002; TDC: r_s_ = -0.34, *p* = 0.009), LP_NonLes_-LP_Les_ (PVI: r_s_ = -0.48, *p* = 0.007), and LP_Les_-PCC (PVI: r_s_ = -0.43, *p* = 0.02) seed pairs were negatively correlated with age. FC values for all other seed pairs were not associated with age, however age was included as a covariate in all subsequent ANCOVAs.

### Functional connectivity within networks

#### Laterality

**Fig. 3:**
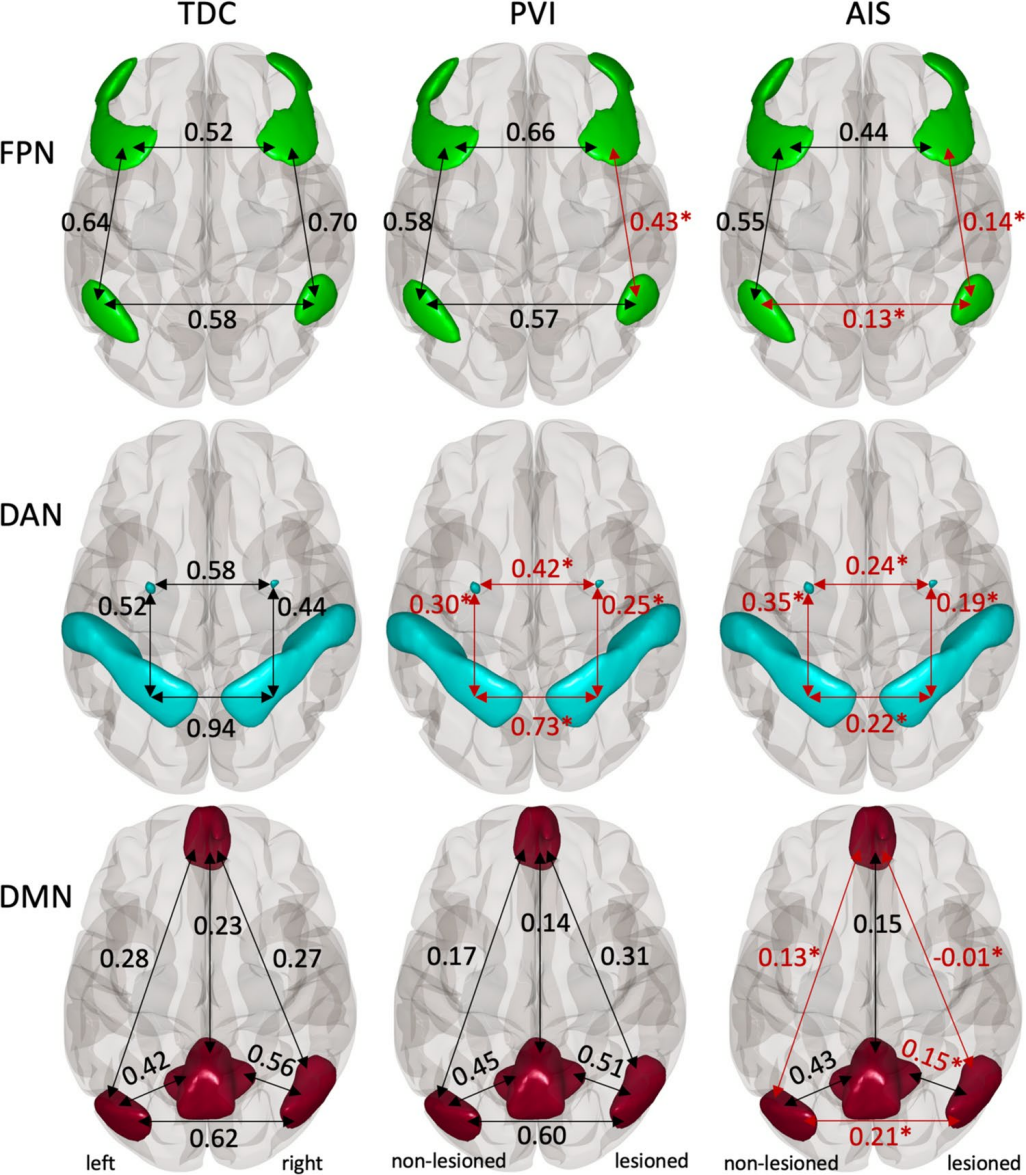
Functional connectivity (FC) was different between groups such that the AIS and PVI groups demonstrated lower FC values compared to TDC for both inter- and intra-hemispheric connectivity. Shown are group mean Fisher-transformed Pearson correlation coefficients for the FPN, DAN and DMN networks for TDC, PVI, and AIS. PVI—Periventricular venous infarction, AIS—Arterial ischemic stroke, FPN—Frontoparietal Network, DAN—Dorsal Attention Network, DMN—Default Mode Network. Red text: *p_FDR_ < 0.05 for the TDC > AIS and TDC > PVI group contrasts. All stroke lesions are on the right side of the brain images

Group mean FC values for each patient group (TDC, PVI, AIS) for each network (FPN, DAN, DMN) are illustrated in Fig. 3. TDC participants showed strong and symmetrical inter- and intra-hemispheric FC values between seed pairs for the DAN and FPN networks and somewhat lower FC values for parts of the DMN. Both AIS and PVI groups showed asymmetrical FC values in the DAN and FPN networks such that intra-hemispheric connectivity values in the lesioned hemisphere were lower than those for the non-lesioned hemisphere. Specifically, the AIS group showed higher FC values (in the FPN) for the LPFC_NonLes_-PPC_NonLes_ seed pair compared to the lesioned pair [t_(24)_ = 7.25, *p* < 0.001, d = 1.45].

For the PVI group, in the FPN, significantly higher FC values were seen for the LPFC_NonLes_-PPC_NonLes_ seed pair compared to the lesioned pair [t_(29)_ = 2.42, *p* = 0.022, d = 0.44]. For the MPFC-LP (in the DMN), higher FC values were found for the lesioned hemisphere over the non-lesioned [t_(29)_ = -3.7, *p* < 0.001, d = 0.68].

#### Group differences

**Fig. 4:**
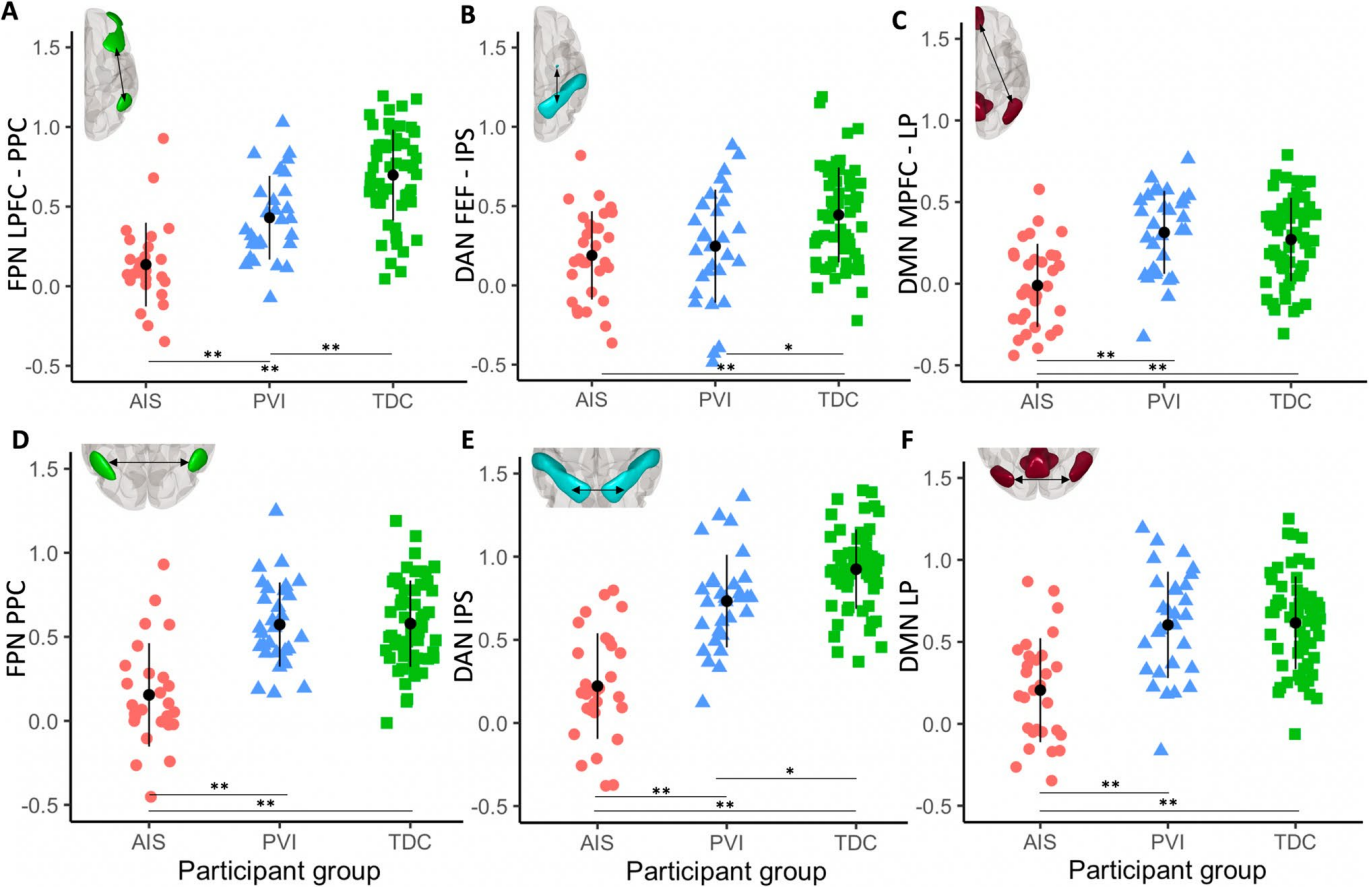
Scatterplots illustrating intra-hemispheric (**A**-**C**) and inter-hemispheric (**D**-**F**) within-network functional connectivity (FC) in three participant groups. (**A**) FC between the lesioned lateral prefrontal (LPFC) and posterior parietal cortex (PPC) seeds in the frontoparietal network (FPN). (**B**) FC between the lesioned frontal eye field (FEF) and intraparietal sulcus (IPS) of the Dorsal Attention Network (DAN). (**C**) FC between the medial prefrontal (MPFC) and lesioned lateral parietal (LP) cortices of the Default Mode Network (DMN). Inter-hemispheric FC values for the (**D**) Posterior parietal cortex (PPC) in the FPN, (**E**) Intraparietal sulci (IPS) in the DAN, and (**F**) Lateral parietal cortices (LP) in the DMN. AIS—Arterial ischemic stroke, PVI—Periventricular venous infarction, and TDC—Typically developing controls. All stroke lesions are on the right side of the brain images. *p_FDR_ < 0.05, **p_FDR_ < 0.001

Significantly higher FC values for numerous seed pairs within all three networks were found for the TDC group compared to the AIS group (Table S1). These differences were found both for intra-hemispheric connectivity values between seed pairs within the lesioned hemisphere, and for several inter-hemispheric seed pairs in the DAN, FPN and DMN (Figs. 3 and 4). No seed pairs for the AIS group showed higher FC values compared to TDC.

Differences were also found between the TDC group and the PVI group (Table S2, Figs. 3 and 4), such that TDC showed higher FC values for seed pairs in the DAN and FPN. No significant differences in FC were found between these two groups in the DMN. Significant differences were found between the AIS and PVI groups, with the PVI group having higher connectivity values within all three networks (Table S3, Fig. 4).

### Functional connectivity between networks

**Fig. 5:**
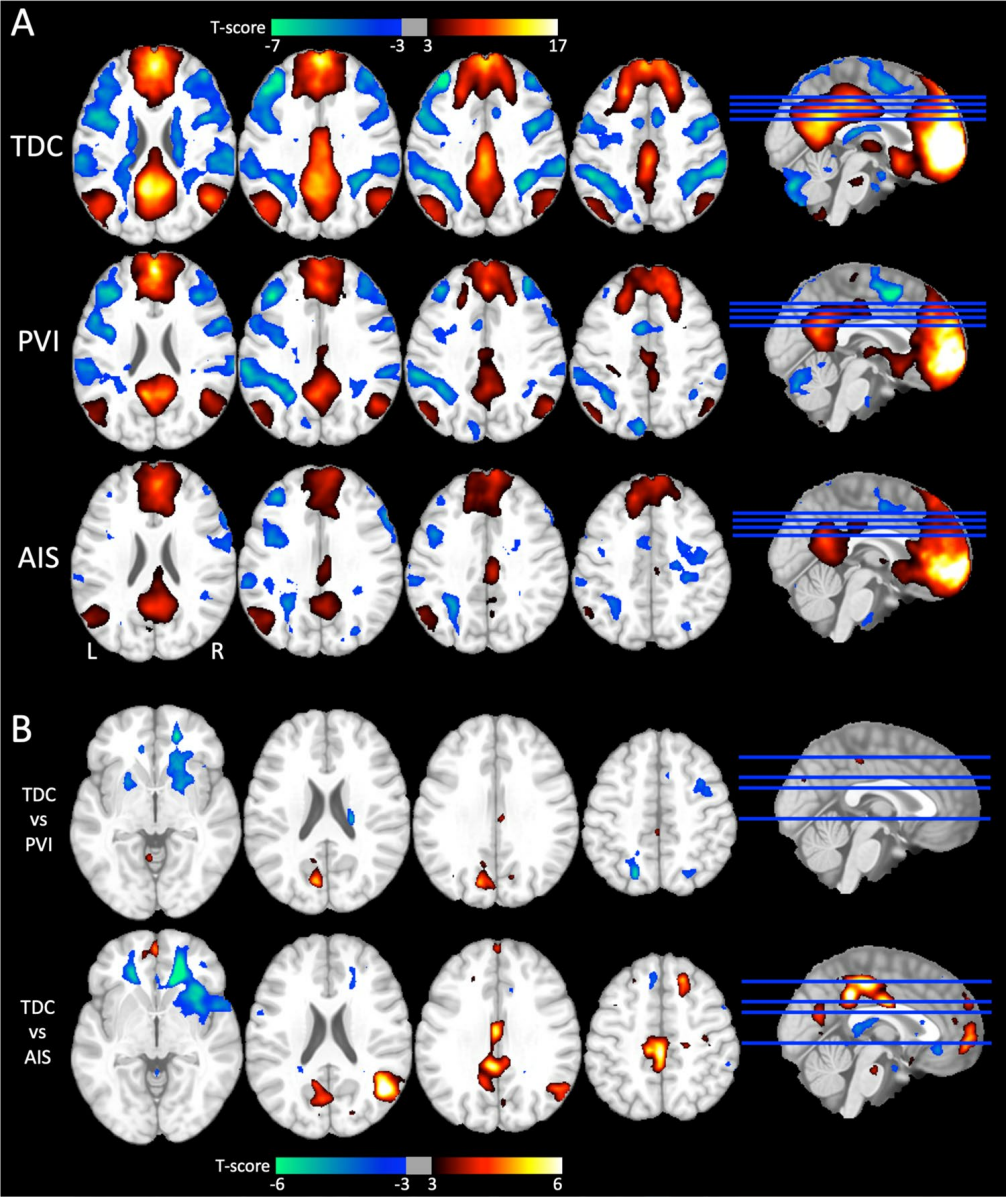
Seed-to-voxel analyses using the medial prefrontal cortex (MPFC) from the DMN as a seed displayed on an MNI template brain. (**A**) Group mean FC maps for three groups where hot colours represent significant positive FC and cool colours represent negative FC (expressed as t-scores). Strong anticorrelations between the seed (DMN MPFC) and the FPN, DAN networks can be seen for the control group (TDC). Weaker anticorrelations are seen for the PVI and AIS groups. (**B**) Statistical contrasts between groups show areas of higher FC for the TDC group compared to the AIS and PVI groups (hot colours) and areas of lower FC in the TDC group compared to both AIS and PVI (cool colours). FC – functional connectivity, AIS—Arterial ischemic stroke, PVI—Periventricular venous infarction, TDC—typically developing controls, DMN—default mode network, FPN—Frontoparietal network, DAN—Dorsal attention network. All stroke lesions are on the right side of the brain images

Seed-to-voxel analyses, using the DMN MPFC as a seed, revealed significant positively correlated voxels (within the DMN) and anticorrelated voxels (within the FPN and DAN) for the TDC group (illustrated in Fig. 5A). Somewhat weaker correlations and anticorrelations were present for the PVI and AIS groups, most notably in the lesioned hemisphere. Voxel-wise statistical contrasts showed that the AIS group had significantly lower positive FC in the LP_Les_ and PCC compared to TDC (Fig. 5B) and PVI. The PVI group showed areas of significantly lower positive FC in the PCC compared to TDC.

### ADHD symptoms, executive function and functional connectivity within networks

Mean measures of executive function and ADHD ratings (Table 2), were not different between AIS and PVI groups (all ps > 0.19), but were different from expected values (BRIEF T-score = 50, ADHD percentile = 50) for many subscales. Age showed associations with executive function on two subscales (after FDR both are not significant: Monitor r_s_ = 0.58, *p* = 0.005; Metacognition r_s_ = 0.46, *p* = 0.026). Age was not associated with ADHD ratings.

**Table 2:** Cognitive function by participant group

Cognitive scores				
BRIEF mean T-scores (SD) [range]	AIS (*N* = 17)	PVI (*N* = 5)	All Stroke (*N* = 22)	Difference from expected
Global Executive Composite (GEC)	59.9 (11.9) [36–77]	61.8 (10.9) [49–72]	60.3 (11.4) [36–77]	t_21_ = 4.2, *p* < 0.001 **
Inhibit	53.4 (11.3) [37–79]	59.0 (5.7) [53–66]	54.7 (10.4) [37–79]	t_21_ = 2.1, *p* = 0.047 *
Shift	57.7 (11.5) [39–72]	63.2 (19.4) [36–84]	59.0 (13.4) [36–84]	t_21_ = 3.2, *p* = 0.005 **
Emotional Control	59.1 (11.8) [38–82]	60.4 (11.9) [43–75]	59.4 (11.6) [38–82]	t_21_ = 3.8, *p* = 0.001 **
Behavioral Regulation Index (BRI)	57.8 (12.0) [39–81]	62.0 (11.5) [46–75]	58.7 (11.8) [39–81]	t_21_ = 3.5, *p* = 0.002 **
Initiate	58.1 (15.3) [35–86]	59.8 (12.8) [44–78]	58.5 (14.5) [35–86]	t_21_ = 2.8, *p* = 0.012 *
Working Memory	62.3 (14.5) [42–89]	60.8 (8.8) [51–72]	62.0 (13.2) [42–89]	t_21_ = 4.2, *p* < 0.001 **
Plan/Organize	60.5 (13.1) [38–89]	60.2 (10.0) [46–73]	60.4 (12.3) [38–89]	t_21_ = 4.0, *p* < 0.001 **
Organization of Materials	52.6 (11.9) [34–71]	54.0 (12.6) [37–67]	53.0 (11.8) [34–71]	t_21_ = 1.2, *p* = 0.252
Monitor	57.7 (12.4) [33–77]	57.8 (12.7) [42–72]	57.7 (12.2) [33–77]	t_21_ = 3.0, *p* = 0.007 **
Metacognition Index (MCI)	60.3 (14.5) [35–89]	60.6 (10.7) [46–71]	60.0 (13.3) [35–89]	t_21_ = 3.6, *p* = 0.002 **
ADHD-5 mean percentiles (SD) [range]	AIS (*N* = 18)	PVI (*N* = 6)	All Stroke (*N* = 24)	
Inattention	72.3 (26.7) [5–99]	66.5 (26.1) [38–95]	70.9 (26.1) [5–99]	W_23_ = 256, *p* = 0.003 **
Hyperactivity	58.3 (30.3) [12–97]	76.0 (15.2) [63–96]	62.8 (28.1) [12–97]	W_23_ = 209, *p* = 0.032 *
Total	67.7 (27.5) [17–99]	73.0 (18.7) [50–96]	69.0 (25.3) [17–99]	W_23_ = 238, *p* = 0.002 **

BRIEF and ADHD scores are negatively scored such that higher values mean poorer performance. *AIS* arterial ischemic stroke, *PVI* periventricular venous infarction, *TDC* typically developing controls, *BRIEF* behaviour rating inventory of executive function, *ADHD-5* ADHD rating scale version 5. All stroke distributions were compared against expected population values for typically developing children: BRIEF T-score = 50, ADHD percentile = 50

^*^p < 0.05, **p < 0.01

**Fig. 6:**
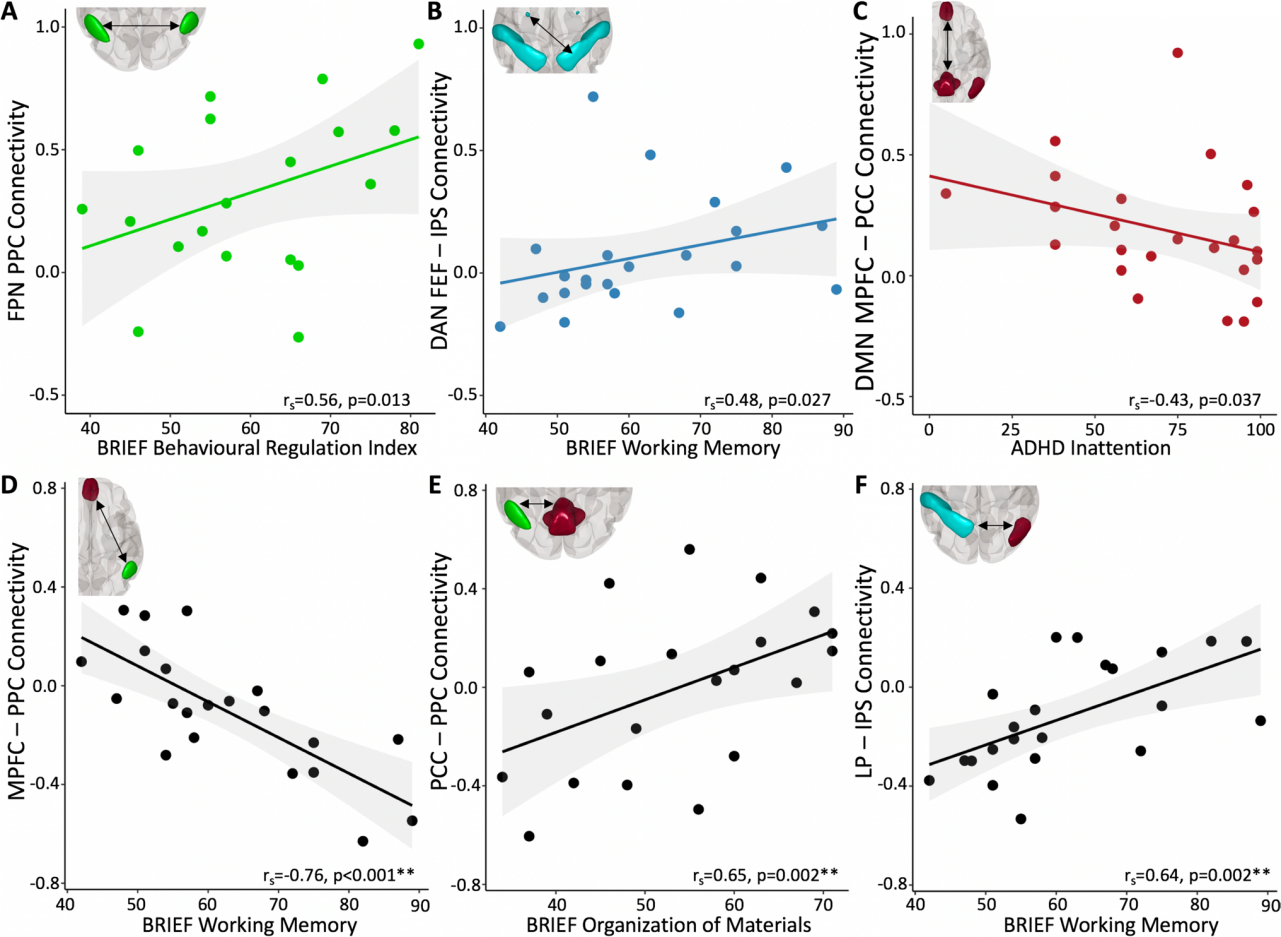
Strongest associations between within-network (**A**-**C**) and between network (**D**-**F**) functional connectivity (FC) and cognitive function. (**A**) Higher FC between the lesioned and non-lesioned Posterior Parietal Cortices (PPC) (in the FPN) was associated with poorer parental ratings on the BRIEF Behavioural Regulation Index. (**B**) Higher FC between the non-lesioned Frontal Eye Field (FEF) and lesioned Intraparietal Sulcus (IPS) (in the DAN) was associated with poorer ratings of working memory. (**C**) Higher FC between the Medial Prefrontal (MPFC) and Posterior Cingulate cortices (PCC) (in the DMN) was associated with better ratings of ADHD Inattention. (**D**) Higher FC between the Medial Prefrontal Cortex (MPFC) (in the DMN) and the lesioned Posterior Parietal Cortex (PPC) (in the FPN) was associated with better parental ratings on the BRIEF Working Memory subscale. (**E**) Higher FC between the Posterior Cingulate Cortex (PCC) (in the DMN) and the non-lesioned Posterior Parietal Cortex (PPC) (in the FPN) was associated with poorer scores on the Organization of Materials subscale. (**F**) Higher FC between the Lateral Parietal Cortex (LP) (in the DMN) and the non-lesioned Intraparietal Sulcus (IPS) (in the DAN) was associated with better ratings of Working Memory. FPN—Frontoparietal Network, DAN—Dorsal Attention Network, DMN—Default Mode Network, r_s_—Spearman’s rho. All stroke lesions are on the right side of the brain images. **p_FDR_ < 0.01. Shaded areas denote the 95% confidence intervals

All associations (controlling for age) between cognitive function and FC measures are shown in Table S4. Higher connectivity values in the FPN were associated with poorer scores on the BRIEF Emotional Control subscale (r_s_ = 0.55, *p* = 0.014) and Behavioural Regulation Index (r_s_ = 0.56, *p* = 0.013, Fig. 6). Higher connectivity values between DAN FEF_NonLes_ and IPS_Les_ in the lesioned hemisphere showed associations with more symptoms of ADHD (r_s_ = 0.45, *p* = 0.031). Associations between poorer cognitive function (Initiate and Working Memory) were also found for higher connectivity values in the DAN. In the DMN, ADHD rating scores showed associations with FC between the MPFC and PCC (r_s_ = -0.43, *p* = 0.037) such that higher connectivity was associated with fewer ADHD symptoms. After FDR correction for multiple comparisons, associations between cognitive function and FC measures were not statistically significant.

### ADHD symptoms, executive function and functional connectivity between networks

Parent ratings of ADHD symptoms and executive function were highly associated with FC values between networks (Table S5, Fig. 6). FC between the DMN MPFC and the FPN PPC_Les_ were highly negatively correlated with many of the BRIEF subscales and ADHD ratings such that higher connectivity values were associated with better function. By contrast, FC values between the DMN PCC and the FPN PPC_NonLes_ were highly positively correlated, such that higher FC was associated with poorer parental ratings of function on both the BRIEF and the ADHD Rating Scale.

## Discussion

We measured RS FC in controls and in two groups of children with perinatal stroke using previously well-established DAN, FPN, and DMN seeds and explored associations with ADHD symptoms and executive function. Compared with typically developing peers, the AIS group showed significantly lower FC values for all networks investigated, most notably in the lesioned hemisphere. The PVI group also showed lower FC in the FPN and DAN networks than controls. Group differences in between-network FC for both stroke groups also demonstrated weaker anticorrelations between task-positive (FPN, DAN) and task-negative (DMN) networks compared to controls. Both within-network and between-network functional connectivity values were highly associated with parental reports of ADHD symptom severity and executive dysfunction symptoms. These results suggest that complex differences in functional connectivity exist both within and between networks after perinatal stroke, which is associated with ADHD and executive function. Investigating methods of altering such functional connectivity could provide insight into pharmacological, cognitive, and modulatory interventions to improve daily living and academic achievement in affected children.

### Within networks

We have shown symmetrical FC between hemispheres in our control group for all three networks investigated (FPN, DAN, DMN). However, in the two stroke groups, intra-hemispheric functional connectivity values were significantly lower within the lesioned compared to non-lesioned hemispheres. We also found lower inter-hemispheric FC in the AIS group compared to controls consistent with past findings in a group of children after childhood arterial stroke (Kornfeld et al., 2018). This finding contrasts a study demonstrating higher FC in the posterior portions of the DMN of children with perinatal arterial stroke (Ilves et al., 2016). Despite relatively consistent and predictable lesions confined to periventricular white matter, our PVI group also showed lower FC values than controls in the FPN and DAN, though past research has not found these same differences (Ilves et al., 2016). These apparent disparities between studies could be due to differences in sample size, lesion size and placement between the two samples and/or differences in the use of voxel-wise contrasts and ROI-based contrasts. Specifically, the Ilves study used probabilistic independent component analysis (PICA), a data-driven method particularly suited to extracting resting state networks from patterns in underlying BOLD signal fluctuations. Our present study used a priori hypotheses to identify atlas-based regions of interest within which we anticipated group differences as well as a seed-to-voxel technique exploring between network anti-correlations. These techniques are appropriate and powerful and the resulting DMN network appears similar in both studies, however have resulted in opposite patterns of group differences between AIS and controls. It is possible that the ICA analysis (Ilves et al., 2016), given its data-driven approach has revealed group differences that lie outside of the regions of interest used in the current study, however such differences would have been detected in our seed-to-voxel based analysis if they existed in our sample. Disparities could also be due to differences in stroke characteristics in the patient samples and differing statistical power due to different sample sizes. Future, more well-powered studies will likely elucidate this disparity.

These findings are not surprising given the presence of cortical, subcortical, and white matter lesions in these groups. Specifically, while lesion sizes and locations were heterogeneous, children in the AIS group all had unilateral damage to brain areas supplied by the middle cerebral artery, and children in the PVI group had unilateral damage to periventricular white matter. Since ROIs in patients disproportionately affected by direct lesion damage were excluded from this study, it is likely that more indirect damage to remote brain areas also mediates FC. Secondary degeneration of remote brain areas structurally connected to the primary lesion, termed diaschisis (Finger et al., 2004; von Monakow, 1914), likely contributed to the laterality favouring FC in the non-lesioned hemisphere. Diaschisis has been previously demonstrated in remote structures in children following early perinatal brain injury (Craig et al., 2019a, b; Rajapakse et al. 2009; Srivastava et al., 2019) as well as in later childhood stroke (Kirton et al., 2016) showing associations with motor function though, thus far, associations with cognitive function has not been extensively addressed (Srivastava et al., 2019).

In perinatal stroke and hemiparetic cerebral palsy, such laterality favouring FC in the non-lesioned hemisphere has previously been found in the motor system of children (Manning et al., 2015, 2016; Saunders et al., 2019), consistent with evidence from other imaging modalities measuring myelination (Yu et al., 2018), white matter tractography of corticospinal tracts (Dinomais et al., 2015; Hodge et al., 2017; Kuczynski et al., 2018), sensory tracts (Kuczynski et al., 2017), as well as structural volumetrics of the cortex (Li et al., 2012), thalamus (Craig et al., 2019a, b), and cerebellum (Craig et al., 2019a, b). Neuroplasticity of functional lateralization between hemispheres has also been found for language networks in children after perinatal stroke (Carlson et al., 2019; Dick et al., 2013). Recent work using cortical morphometry (Al Harrach et al., 2019; Shinde et al., 2021) and graph theory metrics (Craig et al., 2020) have investigated the complementary role of the non-lesioned hemisphere, rather than just the lesioned hemisphere, as a possible compensatory mechanism mediating developmental neuroplasticity. Models of interhemispheric competition in multiple systems, such as motor, somatosensory and language, suggest the presence of extensive neuroplastic mechanisms capable of reorganizing function after early injury (Staudt, 2010a, b). Perhaps returning a system to relative equilibrium and symmetry may enhance function, which may also be true for attention-related networks and others. However, since most research has focussed on motor function in perinatal stroke, there is a relative paucity of literature investigating the effects of excessive laterality on attention-related FC networks.

### Associations with function

In the DMN, higher FC values between frontal and posterior midline regions were associated with lower ADHD symptom parental ratings, consistent with our initial hypothesis. By contrast, we also found that degree of inter-hemispheric FC in the stroke groups was associated with higher symptom ratings such that within the DAN and FPN networks, higher connectivity between lesioned and non-lesioned hemispheres was linked to poorer ADHD symptoms and parent ratings of executive function. This finding was in the opposite direction to our initial hypothesis that greater FC would be associated with better function. While this may seem counterintuitive, it suggests that stronger connectivity between intact and damaged brain areas (whether intra- or inter-hemispheric) may be maladaptive. This is consistent with studies examining connectivity in language and motor systems in children, adolescents and young adults (Dick et al., 2013; Eng et al., 2018). Perhaps a reduction of FC between non-lesioned and lesioned brain areas would be a more adaptive mechanism and a possible future target for rehabilitation and neuromodulation.

### Between networks

We additionally found striking differences in FC between networks as illustrated by strong anticorrelations between the task-negative DMN and task-positive PFN and DAN networks in the control group, and significantly weaker anticorrelations between networks in the AIS and PVI groups. Previous comparisons of FC between networks in ADHD groups have shown a similar pattern of disruption in such anticorrelations in both children and adults (Fassbender et al., 2009; Gao et al., 2019; Uddin et al., 2008), which may even be specific to ADHD subtype (Fair et al., 2012; Tomasi & Volkow, 2012). In our sample, it seems that underlying stroke-induced injuries may also disrupt between-network anticorrelations after perinatal stroke. Further, this finding was highly associated with parental ratings of ADHD symptoms and executive function. Specifically, lower FC (i.e., stronger anticorrelations) between *anterior* regions in the DMN (MPFC) and posterior regions in the FPN (PPC) was associated with poorer function on many subscales of the BRIEF and ADHD rating scales. By contrast, associations between areas of the *posterior* DMN (PCC, LP) and areas in the FPN (PPC, LPFC, IPS) were highly positively correlated with cognitive function such that lower FC (i.e., stronger anticorrelations) was associated with better function. Taken together, these results suggest that cognitive dysfunction in children with perinatal stroke may be due to disruptions of anticorrelations in frontal areas between task-positive attention-related and task-negative default mode networks. Though we cannot conclude causality between putative stroke-induced disruptions in FC networks and the presence of cognitive dysfunction, the finding that they were very highly and systematically correlated is intriguing. It appears that both the within-network and the complex interplay between task-positive and task-negative networks interact to produce the high functional correlations demonstrated here.

## Implications

Models of the development of cognitive control occurring in later childhood and adolescence may provide a helpful theoretical framework for conceptualizing our findings (Luna et al., 2015). As adolescents mature and brain networks become more adult-like, the effort required to achieve adult levels of performance on inhibitory control tasks appears to decrease though activity in frontal areas implicated in performance monitoring, specifically the dorsal anterior cingulate cortex (dACC) may increase (Ordaz et al., 2013). After perinatal stroke, these developmental trajectories in frontal areas may be disrupted by remote lesions via diaschisis resulting in altered FC within and between the DMN and task-positive attention-related networks, consequently reflected in higher prevalence of ADHD and executive dysfunction. This finding is consistent with a recent study finding diffusivity differences in the frontal white matter of the anterior forceps in a similar sample that also showed correlations with parental ratings of ADHD and executive function (Larsen et al., 2021). Such networks may provide targets for cognitive habilitation and inform interventions using non-invasive neuromodulation. Changes in underlying cortical excitability may modulate the strength of functional networks (and links to other networks) in a therapeutic way. Indeed, pharmacological interventions have found changes in functional connectivity associated with successful treatment for ADHD (Battel et al., 2016; Biskup et al., 2016).

## Limitations

We acknowledge certain limitations of this study. While we achieved a sizable sample for the group-level FC comparisons, a more robust data set would have prospectively-collected cognitive scores for a greater number of participants, including those in the control group thereby maximizing statistical power. We also did not screen for executive functioning or ADHD symptoms in the control group assuming that this group likely contained children with ADHD and executive dysfunction consistent with estimated population base rates of ~ 7% (Thomas et al., 2015). The number of children taking stimulant medications to treat ADHD symptoms (in any participant group) was also not collected. When working with pediatric populations, head motion is a ubiquitous imaging limitation (especially in those with ADHD), resulting in the removal of participants and the de-weighting of individual scan volumes within participants still included though we found no group differences in the number of volumes scrubbed due to outliers. Additionally, head motion is known to preferentially affect long-range FC values compared to short-range FC (Power et al., 2015) and this may have added additional variability. We also used the standard MNI template (152-average) in our analysis that was generated from young adult brains. While this template has performed well with pediatric brains in our experience, a customized pediatric template could have been used instead that potentially could have reduced variability. We could have also used a pediatric version of the network ROI atlas for our seeds if one was commonly available. We used undirected network analysis however directed networks may have been able to additionally quantify effective connectivity rather than just covariation. We studied cortical areas but there is evidence suggesting a role for subcortical structures such as the caudate and putamen (von Rhein et al., 2016), areas often damaged following perinatal arterial ischemic stroke, thus differences in cortical functional connectivity in the AIS group may have been additionally mediated by disruptions in such basal ganglia structures. Children with PVI have largely intact subcortical structures, which is consistent with the observation that they show somewhat similar functional connectivity as TDC in cortical networks such as the DMN.

## Conclusion

In summary, our study has shown that children with perinatal stroke have significantly lower FC within and between the DMN, DAN, and FPN compared to controls. Functional correlations suggest that the degree of FC within and between these networks is associated with executive function and ADHD symptomology. These results may suggest maladaptive interhemispheric connectivity in the stroke population with further investigation needed.

### Supplementary Information

Below is the link to the electronic supplementary material.

Supplementary file1 (DOCX 32 KB)

## Data Availability

Data is available from the corresponding author without undue delay upon reasonable request.
