# Supplementary material for: Executive functioning, ADHD symptoms and resting state functional connectivity in children with perinatal stroke
Source: Brain Imaging Behav. 2023 Dec 1;18(2):263–78. doi: 10.1007/s11682-023-00827-w (PMC11156742; doi:10.1007/s11682-023-00827-w)
Supplement: Supplementary file 1 — Supplementary file1 (DOCX 32 KB) [file 11682_2023_827_MOESM1_ESM.docx]

**Supplementary Data**

**Table S1.** Group comparisons between controls and participants with arterial stroke (contrast: TDC>AIS)

| **Network**  Seed pair | **Group** | **N** | **Mean (SD)** | **P value** | **Difference** | **Effect size (η^2^_p_)** |
| --- | --- | --- | --- | --- | --- | --- |
| **Frontoparietal Network (FPN)** | | | | | | |
| LPFC_NonLes_ - PPC_Les_ | AIS  TDC | 26  59 | 0.125 (0.21)  0.284 (0.25) | 0.010* | 0.159 | 0.08 |
| PPC_NonLes_ - PPC_Les_ | AIS  TDC | 27  59 | 0.130 (0.33)  0.578 (0.26) | <0.001* | 0.448 | 0.35 |
| LPFC_Les_ - PPC_Les_ | AIS  TDC | 26  59 | 0.136 (0.26)  0.698 (0.28) | <0.001* | 0.562 | 0.44 |
| **Dorsal Attention Network (DAN)** | | | | | | |
| FEF_NonLes_ - FEF_Les_ | AIS  TDC | 30  59 | 0.243 (0.24)  0.575 (0.25) | <0.001* | 0.332 | 0.32 |
| FEF_NonLes_ - IPS_NonLes_ | AIS  TDC | 30  59 | 0.351 (0.28)  0.519 (0.35) | 0.022* | 0.168 | 0.06 |
| FEF_Les_ - IPS_NonLes_ | AIS  TDC | 30  59 | 0.195 (0.22)  0.381 (0.27) | <0.001* | 0.186 | 0.12 |
| FEF_NonLes_ - IPS_Les_ | AIS  TDC | 30  59 | 0.083 (0.23)  0.289 (0.29) | <0.001* | 0.206 | 0.13 |
| FEF_Les_ - IPS_Les_ | AIS  TDC | 30  59 | 0.189 (0.28)  0.444 (0.30) | <0.001* | 0.255 | 0.20 |
| IPS_NonLes_ - IPS_Les_ | AIS  TDC | 30  59 | 0.222 (0.32)  0.935 (0.25) | <0.001* | 0.713 | 0.60 |
| **Default Mode Network (DMN)** | | | | | | |
| MPFC - LP_NonLes_ | AIS  TDC | 29  59 | 0.127 (0.32)  0.278 (0.30) | 0.027* | 0.151 | 0.06 |
| MPFC - LP_Les_ | AIS  TDC | 31  59 | -0.010 (0.26)  0.271 (0.26) | <0.001* | 0.281 | 0.26 |
| LP_NonLes_ - LP_Les_ | AIS  TDC | 29  59 | 0.206 (0.32)  0.616 (0.28) | <0.001* | 0.410 | 0.30 |
| LP_Les_ - PCC | AIS  TDC | 31  59 | 0.150 (0.34)  0.559 (0.25) | <0.001* | 0.409 | 0.30 |

Table note: Group comparisons (controlling for age and scanner) between TDC and AIS within the three networks. DMN seeds: Medial Prefrontal Cortex (MPFC), Lateral Parietal (LP), Posterior Cingulate Cortex (PCC). DAN seeds: Frontal Eye Fields (FEF), Intraparietal Sulcus (IPS). FPN seeds: Lateral Prefrontal Cortex (LPFC), Posterior Parietal Cortex (PPC), η^2^_p_ - partial Eta squared. Correlations that survived the FDR correction are denoted with *p_FDR_<0.05.

**Table S2.** Group comparisons between controls and participants with periventricular venous infarction (contrast: TDC>PVI)

| **Network**  Seed pair | **Group** | **N** | **Mean** | **P value** | **Difference** | **Effect size (η^2^_p_)** |
| --- | --- | --- | --- | --- | --- | --- |
| **Frontoparietal Network (FPN)** | | | | | | |
| LPFC_Les_ - PPC_Les_ | PVI  TDC | 30  59 | 0.430 (0.26)  0.698 (0.28) | <0.001* | 0.268 | 0.14 |
| **Dorsal Attention Network (DAN)** | | | | | | |
| FEF_NonLes_ - FEF_Les_ | PVI  TDC | 30  59 | 0.419 (0.19)  0.575 (0.25) | 0.009* | 0.156 | 0.08 |
| FEF_NonLes_ - IPS_NonLes_ | PVI  TDC | 30  59 | 0.304 (0.30)  0.519 (0.35) | 0.003* | 0.215 | 0.10 |
| FEF_NonLes_ - IPS_Les_ | PVI  TDC | 30  59 | 0.168 (0.27)  0.289 (0.29) | 0.042* | 0.121 | 0.05 |
| FEF_Les_ - IPS_NonLes_ | PVI  TDC | 30  59 | 0.123 (0.35)  0.381 (0.27) | <0.001* | 0.258 | 0.12 |
| FEF_Les_ - IPS_Les_ | PVI  TDC | 30  59 | 0.247 (0.36)  0.444 (0.30) | 0.019* | 0.197 | 0.06 |
| IPS_NonLes_ - IPS_Les_ | PVI  TDC | 30  59 | 0.733 (0.28)  0.935 (0.25) | 0.003* | 0.202 | 0.10 |
| **Default Mode Network (DMN)** | | | | | | |
| MPFC - PCC | PVI  TDC | 30  59 | 0.140 (0.28)  0.230 (0.26) | 0.026 | 0.09 | 0.06 |

Table note: Group comparisons (controlling for age and scanner) between TDC and PVI within the three networks. DMN seeds: Medial Prefrontal Cortex (MPFC), Lateral Parietal (LP), Posterior Cingulate Cortex (PCC). DAN seeds: Frontal Eye Fields (FEF), Intraparietal Sulcus (IPS). FPN seeds: Lateral Prefrontal Cortex (LPFC), Posterior Parietal Cortex (PPC), η^2^_p_ - partial Eta squared. Correlations that survived the FDR correction are denoted with *p_FDR_<0.05.

**Table S3.** Group comparisons between participants with periventricular venous infarction and arterial ischemic stroke (contrast: PVI>AIS)

| **Network**  Seed pair | **Group** | **N** | **Mean (SD)** | **P value** | **Difference** | **Effect size (η^2^_p_)** |
| --- | --- | --- | --- | --- | --- | --- |
| **Frontoparietal Network (FPN)** | | | | | | |
| LPFC_Les_ - PPC_Les_ | AIS  PVI | 26  30 | 0.136 (0.26)  0.430 (0.26) | <0.001* | 0.294 | 0.26 |
| LPFC_NonLes_ - LPFC_Les_ | AIS  PVI | 27  30 | 0.439 (0.28)  0.656 (0.26) | 0.008* | 0.217 | 0.13 |
| LPFC_NonLes_ - PPC_Les_ | AIS  PVI | 26  30 | 0.125 (0.21)  0.303 (0.25) | 0.005* | 0.178 | 0.14 |
| PPC_NonLes_ - PPC_Les_ | AIS  PVI | 27  30 | 0.130 (0.33)  0.573 (0.25) | <0.001* | 0.443 | 0.36 |
| **Dorsal Attention Network (DAN)** | | | | | | |
| FEF_NonLes_ - FEF_Les_ | AIS  PVI | 30  30 | 0.243 (0.24)  0.419 (0.19) | 0.004* | 0.176 | 0.14 |
| IPS_NonLes_ - IPS_Les_ | AIS  PVI | 30  30 | 0.222 (0.32)  0.733 (0.28) | <0.001* | 0.511 | 0.43 |
| **Default Mode Network (DMN)** | | | | | | |
| MPFC - LP_Les_ | AIS  PVI | 31  30 | -0.010 (0.26)  0.314 (0.25) | <0.001* | 0.322 | 0.29 |
| LP_NonLes_ - LP_Les_ | AIS  PVI | 29  30 | 0.206 (0.32)  0.604 (0.32) | <0.001* | 0.358 | 0.28 |
| LP_Les_ - PCC | AIS  PVI | 31  30 | 0.150 (0.34)  0.506 (0.35) | <0.001* | 0.364 | 0.21 |

Table note: Group comparisons (controlling for age and scanner) between PVI and AIS within the three networks. DMN seeds: Medial Prefrontal Cortex (MPFC), Lateral Parietal (LP), Posterior Cingulate Cortex (PCC). DAN seeds: Frontal Eye Fields (FEF), Intraparietal Sulcus (IPS). FPN seeds: Lateral Prefrontal Cortex (LPFC), Posterior Parietal Cortex (PPC), η^2^_p_ - partial Eta squared. Correlations that survived the FDR correction are denoted with *p_FDR_<0.05.

**Table S4.** Correlations between cognition and functional connectivity within networks.

| **Network**  Seed pair | **N** | **Cognitive outcome** | **Correlation (r_s_)** | **p-value** |
| --- | --- | --- | --- | --- |
| **Frontoparietal Network (FPN)** | | |  |  |
| PPC_NonLes_ - PPC_Les_ | 20 | Emotional Control | 0.552 | 0.014 |
|  | 20 | Behavioural Regulation Index | 0.559 | 0.013 |
| **Dorsal Attention Network (DAN)** | | |  |  |
| FEF_NonLes_ - FEF_Les_ | 22 | Initiate | 0.452 | 0.039 |
| FEF_NonLes_ - IPS_Les_ | 22 | Working Memory | 0.480 | 0.027 |
|  | 24 | ADHD Inattention | 0.452 | 0.031 |
| **Default Mode Network (DMN)** | | |  |  |
| MPFC - PCC | 24 | ADHD Inattention | -0.428 | 0.037 |

Table note: Correlations between cognitive outcomes and functional connectivity (controlling for age) in the Frontoparietal Network (FPN) Dorsal Attention Network (DAN) and Default Mode Network (DMN). Seeds: Medial Prefrontal Cortex (MPFC), Posterior Cingulate Cortex (PCC), Frontal Eye Fields (FEF), Intraparietal Sulcus (IPS), Posterior Parietal Cortex (PPC). Correlations that survived the FDR correction are denoted with *p_FDR_<0.05.

**Table S5.** Correlations between cognition and functional connectivity between networks.

| **Network pair**  Seed pair | **N** | **Cognitive outcome** | **Correlation (r_s_)** | **p-value** |
| --- | --- | --- | --- | --- |
| **Default Mode (DMN) and Frontoparietal Network (FPN)** | | |  |  |
| MPFC - PPC_Les_ | 21 | Global Executive Composite | -0.589 | 0.006* |
|  | 21 | Initiate | -0.489 | 0.029* |
|  | 21 | Working Memory | -0.760 | <0.001* |
|  | 21 | Plan/Organize | -0.576 | 0.008* |
|  | 21 | Organization of Materials | -0.493 | 0.027* |
|  | 21 | Metacognition Index | -0.535 | 0.012* |
|  | 21 | Monitor | -0.488 | 0.029* |
|  | 23 | ADHD Inattention | -0.618 | 0.002* |
|  | 23 | ADHD Hyperactivity | -0.668 | <0.001* |
|  | 23 | ADHD Total | -0.687 | <0.001* |
| PCC - PPC_NonLes_ | 21 | Global Executive Composite | 0.453 | 0.045 |
|  | 21 | Initiate | 0.474 | 0.035 |
|  | 21 | Working Memory | 0.528 | 0.017* |
|  | 21 | Plan/Organize | 0.552 | 0.012* |
|  | 21 | Organization of Materials | 0.645 | 0.002* |
|  | 21 | Metacognition Index | 0.532 | 0.013* |
|  | 23 | ADHD Inattention | 0.548 | 0.008* |
|  | 23 | ADHD Total | 0.454 | 0.034 |
| PCC - LPFC_NonLes_ | 20 | Plan/Organize | 0.568 | 0.011 |
|  | 20 | Organization of Materials | 0.529 | 0.020 |
|  | 22 | ADHD Inattention | 0.451 | 0.040 |
| **Default Mode (DMN) and Dorsal Attention Network (DAN)** | | |  |  |
| LP_Les_ - IPS_NonLes_ | 22 | Global Executive Composite | 0.462 | 0.035 |
|  | 22 | Initiate | 0.450 | 0.040 |
|  | 22 | Working Memory | 0.638 | 0.002* |
|  | 22 | Plan/Organize | 0.614 | 0.003* |
|  | 22 | Metacognition Index | 0.536 | 0.010* |
|  | 24 | ADHD Inattention | 0.429 | 0.041 |
|  | 24 | ADHD Total | 0.472 | 0.023 |

Table note: Correlations between cognitive outcomes and functional connectivity (controlling for age) between networks. DMN seeds: Medial Prefrontal Cortex (MPFC), Lateral Parietal (LP), Posterior Cingulate Cortex (PCC). DAN seeds: Frontal Eye Fields (FEF), Intraparietal Sulcus (IPS). FPN seeds: Lateral Prefrontal Cortex (LPFC), Posterior Parietal Cortex (PPC), *p_FDR_<0.05.
